# Development of a new TNM staging system for poorly differentiated thyroid carcinoma: a multicenter cohort study

**DOI:** 10.3389/fendo.2025.1586542

**Published:** 2025-08-19

**Authors:** Yu Guo, Qianyi Zhong, Liangen Xie, Xudong Wang, Liang Guo, Shimin Zhuang, Chunyan Jia, Lijiao Wu, Jin Peng, Feng Pang, Ankui Yang, Tianrun Liu

**Affiliations:** ^1^ Department of General Surgery (Thyroid Surgery), The Sixth Affiliated Hospital, Sun Yat-sen University, Guangzhou, China; ^2^ Department of Otorhinolaryngology Head and Neck Surgery, The Sixth Affiliated Hospital, Sun Yat-sen University, Guangzhou, China; ^3^ Biomedical Innovation Center, The Sixth Affiliated Hospital, Sun Yat-sen University, Guangzhou, China; ^4^ Department of Thyroid Surgery, Sun Yat-sen Memorial Hospital, Sun Yat-sen University, Guangzhou, China; ^5^ The First Clinical Medical College of Jinan University, Guangzhou, Guangdong, China; ^6^ Department of Otorhinolaryngology Head and Neck Surgery, Guangzhou Twelfth People’s Hospital (The Affiliated Twelfth People’s Hospital of Guangzhou Medical University), Guangzhou Medical University, Guangzhou, China; ^7^ Department of Maxillofacial and Otorhinolaryngological Oncology, Tianjin Medical University Cancer Institute & Hospital, National Clinical Research Center for Cancer, Tianjin's Clinical Research Center for Cancer, Key Laboratory of Basic and Translational Medicine on Head & Neck Cancer, Tianjin, Key Laboratory of Cancer Prevention and Therapy, Tianjin, Tianjin, China; ^8^ Department of Head and Neck Surgery, Institute of Cancer Research and Basic Medical Sciences of Chinese Academy of Sciences, Cancer Hospital of University of Chinese Academy of Sciences, Zhejiang Cancer Hospital, Hangzhou, China; ^9^ Department of Otolaryngology Head and Neck (Thyroid) Surgery, Shenshan Medical Center, Sun Yat-sen Memorial Hospital, Sun Yat-sen University, Shanwei, China; ^10^ Department of Head and Neck Surgery, Sun Yat-sen University Cancer Center, Guangzhou, China; ^11^ State Key Laboratory of Oncology in South China, Guangzhou, China

**Keywords:** poorly differentiated thyroid cancer, new TNM stage, adjusted hazard ratio, cancer-specific survival, prognosis prediction

## Abstract

**Objective:**

Poorly differentiated thyroid cancer (PDTC) is a rare, heterogeneous carcinoma from follicular cells, characterized by poor differentiation, aggressive spread, and poor prognosis. Currently, there is no specific staging system for PDTC. This study aimed to develop a new TNM staging system tailored to PDTC for improved disease management.

**Methods:**

A new TNM staging system was designed and internally validated using data from the US SEER database (2004-2016) on PDTC cases. External validation was performed using data from four major institutions in China. Prognostic factors influencing cancer-specific survival (CSS) were identified through Cox regression analyses. Patients were stratified into subgroups based on adjusted hazard ratios (AHRs), weighted by these prognostic factors. The new system classified patients into five stages with distinct 5-year CSS outcomes.

**Results:**

The study analyzed 1,201 PDTC cases from SEER and 85 cases from China. Among the 876 patients in the training cohort, the new TNM staging system showed superior discrimination compared to the 8th edition of the AJCC TNM system. The 5-year CSS rates for the new stages I, II, III, IVA, and IVB were 96.3%, 88.4%, 69.4%, 43.3%, and 22.3%, respectively. The new system outperformed the 8th edition in predicting CSS, as shown by time-dependent ROC curves, C-index, and calibration plots. Both internal and external validation confirmed its predictive abilities.

**Conclusion:**

The current AJCC staging system inadequately predicts PDTC prognosis. The new TNM staging system developed in this study offers improved stratification and prognosis prediction, potentially guiding more effective clinical management for PDTC.

## Introduction

Poorly differentiated thyroid carcinoma (PDTC) is an uncommon and heterogeneous type of follicular cell-derived thyroid carcinomas (TC) (1), with an intermediate biological behavior between well-differentiated (papillary and follicular) and undifferentiated (anaplastic) carcinoma (2). The estimated incidence of PDTC is low, accounting for only 1%-3% of all thyroid malignancies (3, 4). PDTC is associated with a more aggressive clinical course than differentiated thyroid carcinoma (DTC), with a higher incidence of late-stage diagnosis, gross extrathyroidal extension (ETE), lymph node involvement, and regional and distant metastases (5–7).

PDTC was recognized as a distinct subtype of thyroid cancer by the World Health Organization (WHO) in 2004 (8). The Turin consensus criteria algorithmic approach, proposed in 2006 (9) and now adopted in the latest WHO classification (10), is used to diagnose PDTC. The most common cause of death associated with non-anaplastic follicular cell-derived TC was PDTC (11). However, the recently adopted edition of the AJCC staging system does not include PDTC as a separate category (12). Most differentiated thyroid cancers (DTC) patients with DTC were highly curable with a 10-year relatively high survival rate of 90% (13), while PDTC was more aggressive and invasive, with a 5-year overall survival rate of 62%-72.8% (7, 11).

According to a study by Agnieszka et al (14), patients with PDTC had a higher mortality rate than those with DTC at comparable stages, indicating that the AJCC 8th staging system may be insufficient for PDTC patients. For clinicians, there is no practical and independent staging standard for assessing PDTC-related death risks.

This study aimed to establish a new TNM staging system for PDTC. We developed new TNM staging criteria for PDTC for better prognosis prediction of PDTC using the SEER database and performed external validation in a large Chinese multicenter database.

## Methods

### Participants and study design

Patients with PDTC were identified using the SEER database from 2004 to 2016. The International Classification of Diseases in Oncology, Third Revision (ICD-O-3) was used to determine the histology codes for PDTC, which included 8050/3, 8260/3, 8290/3, 8330/3, 8331/3, 8332/3, 8335/3, 8337/3, 8340/3, 8341/3, 8342/3, 8343/3, and 8344/3. The primary site was the thyroid gland. The inclusion and exclusion criteria were summarized (**Appendix Figure A1**, online only). To develop a new TNM staging system, a training cohort consisting of patients diagnosed from 2004 to 2013 (n = 876) was used, and the system was validated using an internal validation cohort of patients diagnosed from 2014 to 2016 (n = 325). For research purposes, SEER data are freely accessible.

To validate the external model, we utilized retrospective data from a cohort of 85 patients with PDTC who received treatment at four high-capacity medical institutions in China: The Sixth Affiliated Hospital of Sun Yat-sen University, Sun Yat-sen University Cancer Center, Tianjin Cancer Hospital, and Zhejiang Cancer Hospital. Ethical approval for this study was obtained from the leading unit’s ethics committee of the China multicenter institution. Where obtaining individual patient consent was not feasible, the requirement was waived by the chairperson of the ethics committee. All data used were de-identified and analyzed retrospectively, ensuring respect for patient privacy and upholding ethical standards.

### Statistical analysis

The primary study outcome was cancer-specific survival (CSS), defined as months from cancer diagnosis to death attributable to this cancer. Categorical variables were compared using Fisher’s exact test. To identify significant factors that affect the CSS of PDTC, both univariate and multivariate Cox proportional hazards regression analyses were performed. We formally assessed the proportional hazards assumption using Schoenfeld residual tests for key covariates, including age, T stage, N stage, and M stage. These analyses indicated no significant violations (global Schoenfeld test, *P* = 0.1417), confirming the robustness of the proportional hazards assumption in our Cox regression model. Based on the weight of the selected prognostic factors (age/T/N/M) for staging, PDTC patients were grouped into different subgroups by adjusted hazard ratio (AHR) and referred to as AHR stages (15). The probability of 5-year CSS was assessed for each subgroup and rearranged in ascending order. A new staging system was developed by dividing all subgroups into five sections, with significant differences observed in 5-year CSS.

We utilized the Kaplan-Meier method to calculate cumulative survival time and compared survival curves using the log-rank test. A higher log-rank test statistic indicates a greater distance between survival curves, and its P value indicates statistical significance. Calculations of hazard consistency, hazard discrimination, outcome prediction, and sample size balance were used to compare the two sets of stage groupings based on Groome et al’s original evaluation criteria (16). To validate the ranking of different stage schemas, we calculated bootstrap scores and ranks based on 1,000 bootstrap replications by an online Web server (available in http://rpa.renlab.org) (17).

Cox hazards regression was used to estimate the relationship between the new staging system and AJCC 8th for CSS. The time-ROC and time-dependent C-index was used to quantify the models’ predictive performance, and the calibration plots were used to compare model fits. Predictions should fall on a diagonal line of 45 degrees in a well-calibrated model.

All statistical tests were based on 2-sided probability with significance levels set at *P* < 0.05, and all statistical analysis was performed using the R language (available at URL: www.r-project.org [accessed February 2023]).

## Results

A total of 1286 patients were included in this study, including SEER cohort (n = 1201, median follow-up of 97.9 months) and China cohort (n = 85, median follow-up of 84.7 months). The patient characteristics in the SEER and China cohorts were presented in **Table 1**, and the patient characteristics in the training and internal validation cohorts from the SEER database are described (**Appendix Table 1A**, online only).

**Table 1:** Demographic and clinicopathological characteristics of PDTC patients in the SEER and china cohorts.

Factor	SEER cohort	China cohort	P
	2004-2016 (n = 1201)	external validation (n = 85)	
Age, years			
< 55	512 (42.6%)	50 (59%)	0.005
≥ 55	689 (57.4%)	35 (41%)	
Sex			
Female	718 (59.8%)	44 (52%)	0.18
Male	483 (40.2%)	41 (48%)	
Race			
Asian or Pacific Islander	136 (11.3%)	85 (100%)	< 0.001
Black	99 (8.2%)		
White	952 (79.3%)		
Other	14 (1.2%)		
T stage			
T1	192 (16.0%)	15 (18%)	0.232
T2	219 (18.2%)	16 (19%)	
T3	525 (43.7%)	28 (33%)	
T4a	153 (12.7%)	17 (20%)	
T4b	112 (9.3%)	9 (10%)	
N stage			
N0	807 (67.2%)	20 (23%)	< 0.001
N1a	134 (11.2%)	5 (6%)	
N1b	220 (18.3%)	17 (20%)	
N1NOS	40 (3.3%)	43 (51%)	
M stage			
M0	1054 (87.8%)	64 (75%)	0.002
M1	147 (12.2%)	21 (25%)	
Surgery			
Yes	1171 (97.5%)	79 (93%)	0.034
No	30 (2.5%)	6 (7%)	
Radiation			
Yes	822 (68.4%)	61 (72%)	0.605
No	379 (31.6%)	24 (28%)	
CSS			
Alive	912 (75.9%)	41 (48%)	< 0.001
Dead	289 (24.1%)	44 (52%)	
1-year CSS	93.0%	94%	
3-year CSS	86.2%	80%	
5-year CSS	80.4%	68%	
10-year CSS	71.5%	44%	

CSS, cancer-specific survival.

The distributions of sex, T stage, surgery, and radiotherapy were not significantly different between the SEER and China cohorts (*P* > 0.05). However, compared to patients from the SEER database, patients in China tended to be younger (age < 55 years: 59% vs 42.6% in SEER) and had higher rates of lymph node metastasis (77% vs 32.8% in SEER) and distant metastasis (25% vs 12.2% in SEER) (all *P* < 0.05). The 1-/3-/5-/10-year CSS of PDTC patients from SEER data were 93.0/86.2/80.4/71.5%, respectively, while the 1-/3-/5-/10-year CSS of PDTC patients from China were 94/80/68/44%, respectively. Patients in China tended to have lower rates of CSS (*P* < 0.001).

### Evaluate the prognosis abilities of the AJCC 8th TNM staging system for PDTC

Based on the 8th edition, 5-year CSS for PDTC was 92.8% for stage I (n = 448), 80.1% for stage II (n = 226), 58.3% for stage III (n = 65), 42.4% for stage IVA (n = 55), and 32.4% for stage IVB (n = 82) (**Table 2**). The 5-year CSS showed no significant difference between patients with stage III and IVA (*P* > 0.05; **Table 3**). The lack of a monotonic gradient in the AJCC 8th TNM staging system’s predictive performance for PDTC suggests that its prognostic abilities may be suboptimal.

**Table 2:** Summary of the AJCC 8th and new staging system for PDTC in training cohort.

Stage	Age < 55 years	Age ≥ 55 years		No.	5-year CSS	HR (95% CI)
AJCC 8th staging system						
I	T1-4/N0-1/M0		T1-2/N0/M0	448	92.8%	1 [Reference]
II	M1		T1-2/N1/M0, T3/N0-1/M0	226	80.1%	3.43 (2.41-4.89)
III			T4a/N0-1/M0	65	58.3%	8.04 (5.29-12.23)
IVA			T4b/N0-1/M0	55	42.4%	10.81 (7.09-16.48)
IVB			T1-4/N0-1/M1	82	32.4%	17.13 (11.94 -24.58)
The new staging system						
I	T1/N0-1/M0, T2-3/N0/M0		T1/N0/M0	305	96.3%	1 [Reference]
II	T2-3/N1/M0		T2-3/N0/M0	243	88.4%	3.86 (2.29-6.46)
III	T4/N0-1/M0		T1-3/N1/M0, T4/N0/M0	165	69.4%	8.49 (5.30-13.59)
IVA	M1		T4/N1/M0, T1-3/N0-1/M1	124	43.3%	16.48 (10.30-26.39)
IVB			T4/N0-1/M1	39	22.3%	44.40 (26.79-73.59)

AJCC, American Joint Committee on Cancer; CSS, cancer-specific survival; HR, hazard ratio; CI, confidence interval.

**Table 3:** P values for 5-year CSS and HR on pair-wise stage comparison for the training cohort.

Factor	AJCC 8th staging system		The new staging system	
	*P* for 5-year CSS	*P* for HR	*P* for 5-year CSS	*P* for HR
I vs II	5.31E-12	2.17E-10	2.87E-08	1.42E-07
II vs III	8.78E-06	4.57E-06	1.42E-05	6.20E-06
III vs IVA	0.498	0.528	2.66E-06	9.97E-06
IVA vs IVB	0.007	0.018	3.02E-05	2.66E-05

AJCC, American Joint Committee on Cancer; CSS, cancer-specific survival; HR, hazard ratio.

**Figure 1:**
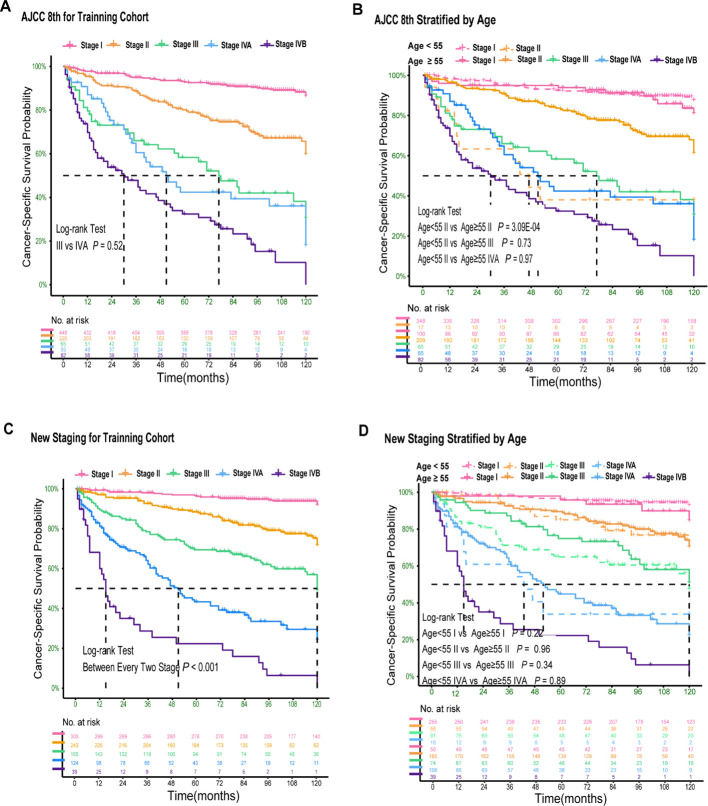
Kaplan–Meier Curves Comparing Cancer-Specific Survival Across the AJCC 8th and New Staging System for PDTC. Kaplan–Meier curves comparing cancer-specific survival across the AJCC 8th staging system **(A)** and stratified by age of 55 years **(B)**; Kaplan–Meier curves comparing cancer-specific survival across the new staging system **(C)** and stratified by age of 55 years **(D)**.

**Figure 1** displays the Kaplan-Meier curves of the patients in the training cohort categorized according to the 8th edition. The 10-year CSS curves indicated no significant disparity between patients with stage III and IVA (*P* > 0.05; **Figure 1A**). Notably, young patients (age < 55 years) with stage II demonstrated a significant difference when compared to older patients (age ≥ 55 years) with stage II (*P* < 0.05; **Figure 1B**). However, no significant differences were observed among older patients with stages III and IVA (*P* > 0.05; **Figure 1B**). The discriminatory abilities of the 8th edition were unsatisfactory as survival differences were observed.

### Establishment and evaluation of a new TNM staging system for PDTC

In the training cohort, we conducted univariate and multivariate Cox regression analyses and found that age, T stage, N stage, and M stage had significant effects on CSS in patients with PDTC (**Appendix Table 2A**, online only). Using the AHRs, we derived 24 subgroups based on age (< 55 or ≥ 55 years), T (T1, T2-3, T4), N (N0, N1), and M (M0, M1) ordinal variables. The 5-year CSS probability of each subgroup was arranged from high to low, and groups with probabilities ranging from 0.90-1, 0.75-0.9, 0.55-0.75, 0.30-0.55, and 0-0.30 were classified as stage I, II, III, IVA, and IVB, respectively (**Appendix Table 3A**, online only).

In the training cohort, univariate and multivariate Cox regression analyses demonstrated that age, T stage, N stage, and M stage were independent predictors of cancer-specific survival (CSS) in PDTC patients (**Appendix Table 2A**, online only). Based on these findings, we initially stratified patients into 24 subgroups according to combinations of age (< 55 vs ≥ 55 years), T classification (T1, T2–3, T4), N status (N0 vs N1), and M status (M0 vs M1). The detailed methodology, including subgroup derivation, statistical evaluation, merging criteria, and explicit CSS probability thresholds for assigning these subgroups into five stages (I–IVB), is comprehensively described in the decision-tree flowchart (**Appendix Figure 2A**, online only). Specifically, the 5-year CSS probability was calculated for each subgroup and ranked from highest to lowest. Based on pre-defined CSS probability thresholds, the subgroups were finally categorized as follows: stage I (CSS probability 0.90–1.00), stage II (0.75–0.90), stage III (0.55–0.75), stage IVA (0.30–0.55), and stage IVB (0.00–0.30) (**Appendix Table 3A**, online only). According to this newly established TNM staging system, the 5-year CSS rates for PDTC patients were 96.3% for stage I (n = 305), 88.4% for stage II (n = 243), 69.4% for stage III (n = 165), 43.3% for stage IVA (n = 124), and 22.3% for stage IVB (n = 39) (**Table 2**). Significant differences in 5-year CSS were observed between all adjacent stages (all P < 0.001; **Table 3**), indicating excellent monotonicity of the CSS gradient in our proposed staging system. Moreover, the 10-year CSS curves showed consistent and significant differentiation between all staging groups (all P < 0.001; **Figure 1C**), without significant differences observed between younger and older patients within the same stage (all P > 0.05; **Figure 1D**). The new staging system demonstrated superior discrimination with larger χ² values of log-rank tests compared to the AJCC 8th edition (**Appendix Table 4A**, online only).

**Figure 2:**
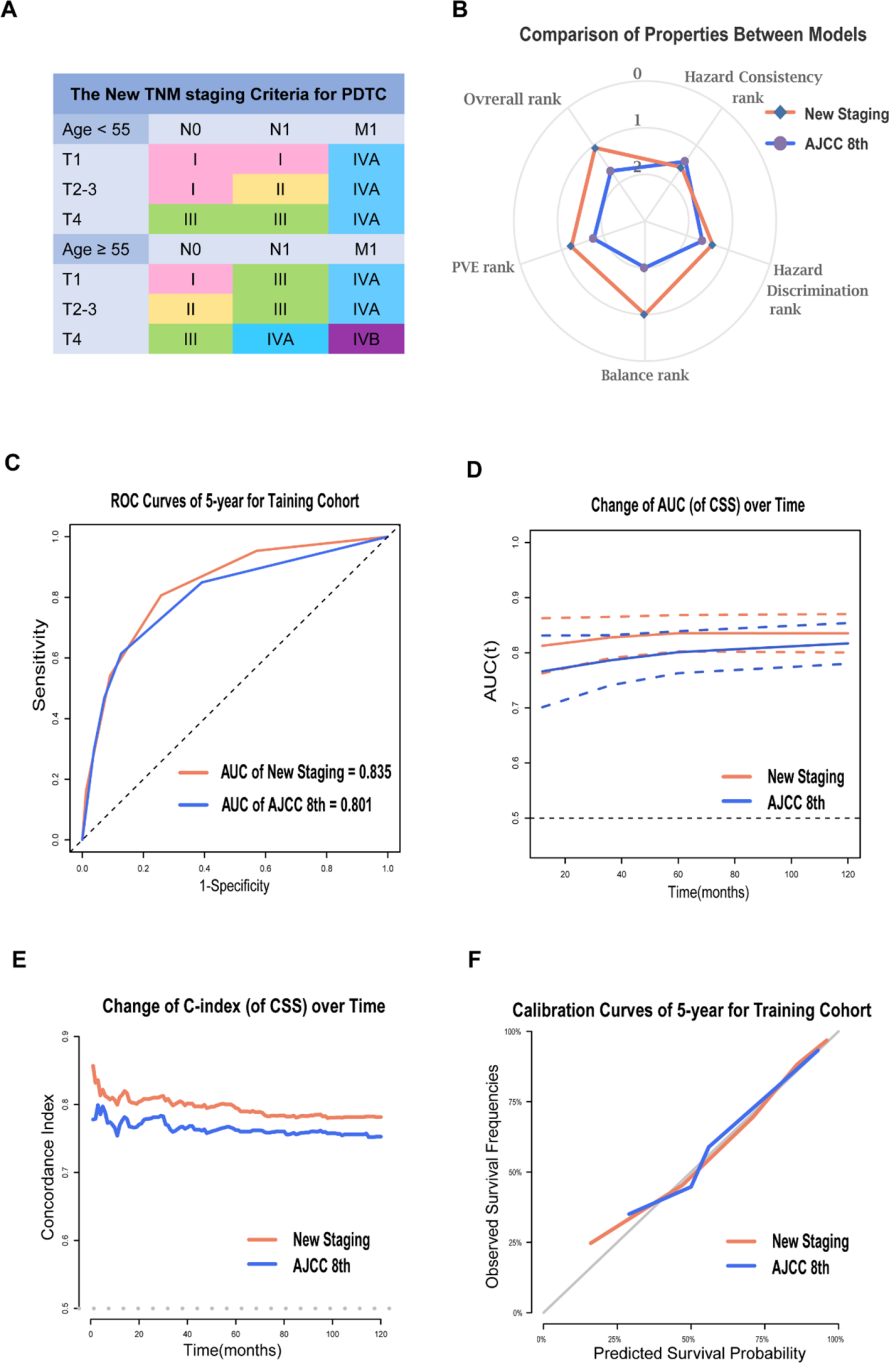
Prognosis Prediction Performances and Validation of the New Staging System for PDTC. The new TNM staging criteria for PDTC **(A)**; The radar map showing the normalized rank of four performance indicators for two models **(B)**; ROC curves of 5-year **(C)**, time-AUC curves **(D)**, time-dependent C-index curves **(E)** and calibration curves of 5-year **(F)** for AJCC 8th and the new staging system in training cohort.

Using stage I as the reference, the hazard ratios of the new staging and the 8th edition were 3.86 (95% CI, 2.29-6.46) vs 3.43 (95% CI, 2.37-4.98) for stage II, 8.49 (95% CI, 5.30-13.59) vs 8.40 (95% CI, 5.29-12.23) for stage III, 16.48 (95% CI, 10.30–26.39) vs 10.81 (95% CI, 7.09–16.48) for stage IVA, and 44.40 (95% CI, 26.79–73.59) vs 17.13 (95% CI, 11.94–24.58) for stage IVB (*P* > 0.05; **Table 2**). Additionally, the hazard ratios for 5-year CSS for the AJCC 8th edition and new staging were presented (**Appendix Figure 3A**, online only). Both the χ2 statistic and hazard ratio for CSS showed better discrimination between every two groups in the new TNM staging than in the AJCC 8th TNM staging (**Table 3**). The new TNM staging criteria are illustrated in **Figure 2A**, and bootstrap validation indicated that the new staging ranked first in hazard discrimination, outcome prediction, and balance (**Appendix Table 5A**, online only). Furthermore, **Appendix Figure 4A** showed that the new TNM staging had better balance, and **Appendix Figure 5A** and **Appendix Table 6A** demonstrated that the new staging had better consistency of hazard ratios of staging subgroups. Overall, the new TNM staging ranked first and outperformed the AJCC 8th edition (**Figure 2B**).

### Prognosis prediction performances and validation of the new TNM staging system for PDTC

The new staging system demonstrated greater predictive power than the 8th edition in the training cohort, with a 5-year AUC of 0.835 compared to 0.801 for 5-year PDTC CSS (**Figure 2C**). The time-AUC (**Figure 2D**) and time-dependent C-index (**Figure 2E**) of the new staging consistently outperformed the 8th edition, indicating that the new TNM staging was superior in predicting PDTC CSS. Moreover, the calibration curve of the new TNM staging displayed better correspondence between predicted and actual outcomes (**Figure 2F**).

**Figure 3:**
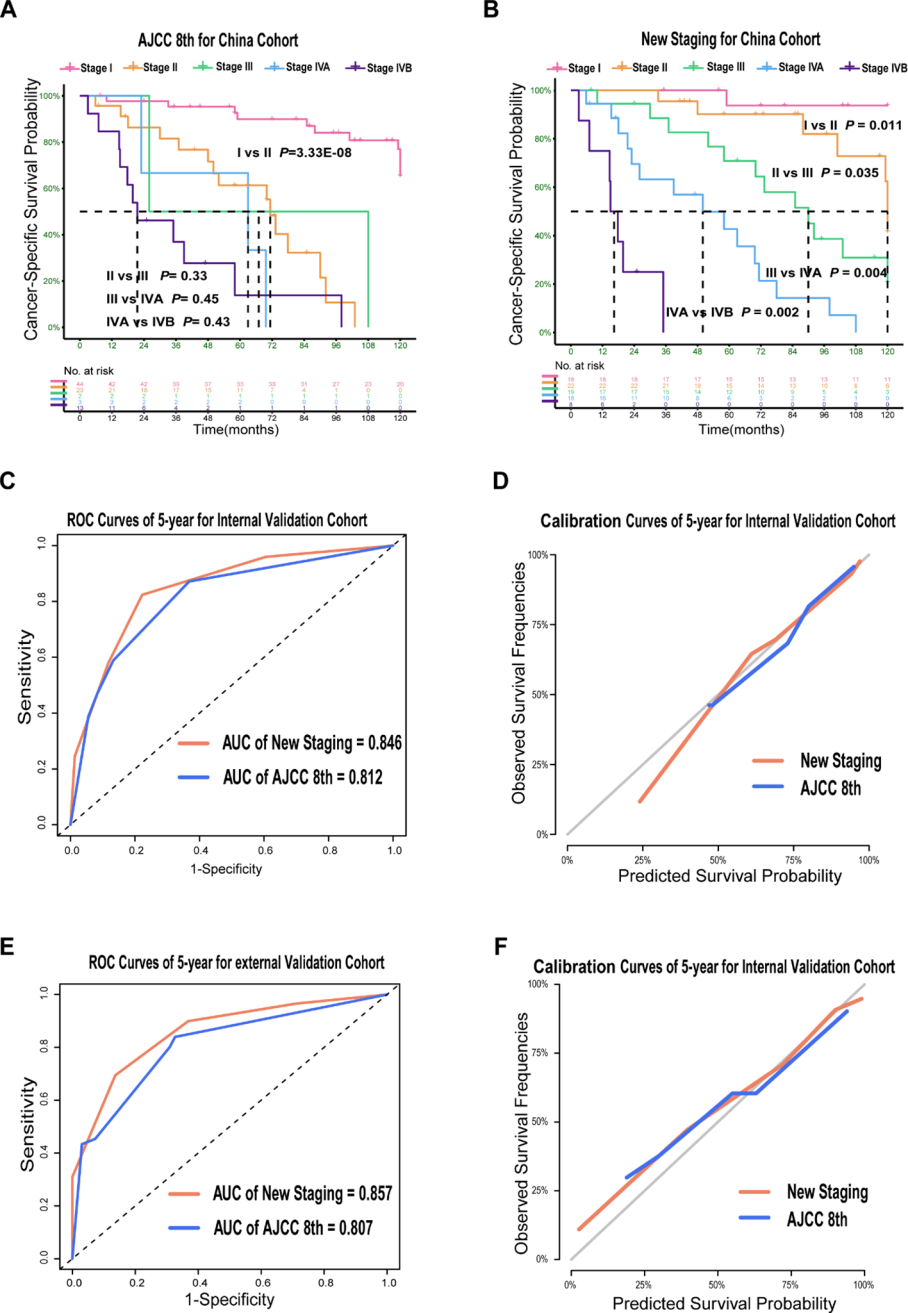
Prognosis Prediction Performances and Validation of the New Staging System for PDTC in Validation Cohort. Kaplan–Meier Curves comparing cancer-specific survival across the AJCC 8th staging system **(A)** and across the new staging system **(B)** in China cohort; ROC curves **(C, E)** and calibration curves **(D, F)** for the AJCC 8th and new staging system in validation cohort.

The new TNM staging system showed superior predictive ability compared to the AJCC TNM staging in all three cohorts. In the training cohort, the new TNM staging had an overall C-index of 0.785, while the AJCC TNM stage had a C-index of 0.759. In the internal validation cohort, the new TNM staging had an overall C-index of 0.835, compared to 0.740 for the 8th edition. In the external validation cohort, the new TNM staging had an overall C-index of 0.815, which was significantly better than the 0.765 observed for the 8th edition. The results from the China cohort revealed that the new TNM staging had better discrimination between every two groups compared to the AJCC 8th TNM staging (**Figures 3A, B**). Furthermore, the new TNM staging exhibited a better overall AUC for CSS prediction than the 8th edition, with a 5-year AUC of 0.846 for the internal cohort (**Figure 3C**) and 0.857 for the external cohort (**Figure 3E**). Finally, the new TNM staging demonstrated better calibration in both the internal and external validation sets (**Figures 3D, F**).

## Discussion

The AJCC 8th staging system lacked the ability to distinguish mortality risk among stages, resulting in homogeneity in prognosis prediction for patients with PDTC. Therefore, a new TNM staging system was developed and validated to improve prognosis prediction stratification. To facilitate clinical application, the age threshold for staging and classifications of the “T” and “N” categories were unchanged. Our focus was on improving prognosis prediction of patient stratification based on CSS, resulting in better discrimination and more evident monotonicity in 5-year CSS rates between every two groups in the new TNM staging system compared to the 8th edition. Additionally, the new TNM staging system demonstrated better accuracy and fitting in all cohorts than the 8th edition.

As the global standard for staging solid tumors, the AJCC’s TNM classification allows for cross-population comparisons regardless of country or ethnicity (18). DTC is unique among cancers in that patient age is part of AJCC TNM staging (19). In the AJCC 8th edition, an age cut-off of 55 years was established in well DTC for risk stratification (12), but the optimal age threshold for staging is an evolving issue. A study by Yan et al (20) suggested that the age cut-off for PTC patients should be 58.5 years and FTC should not be evaluated using the same criteria as those used for PTC. In multivariate analysis adjusting for pathotypes, T, N, and M groups, each difference was statistically significant (21). Due to this, it is impossible to establish an optimal cut point for separating patients at low and high risk of thyroid cancer deaths. In the 8^th^ edition, despite using the same T categories as DTC for consistency, ATC does not consider age when staging (22). However, our study showed that age ≥ 55 years is a crucial factor in predicting a higher risk of thyroid cancer deaths, with a higher HR (2.81) than T2 or N1 in multivariate analysis. Thus, age has been introduced as part of the new staging for PDTC, as patient age was proved to be one of the most important prognostic factors for patients with PDTC.

Metastatic disease (M1) is responsible for the majority of deaths in patients with thyroid cancer, with the five-year survival rate plummeting from almost 99% to below 50% (23). The National Cancer Database (NCDB) cohort study demonstrated that PDTC had a substantial baseline risk of M1 disease, with a prevalence of 17.4%, compared to the 1% prevalence in well-differentiated papillary thyroid carcinoma (WDPTC) (24). Our analysis of SEER and China cohorts found that PDTC had M1 prevalence rates of 12.2% and 25%, respectively. Interestingly, the metastatic nodal burden appeared to have little effect on PDTC with M1 disease in the NCDB cohort. DTC patients with N1a or N1b did not change the prognosis stage by AJCC 8th, and notably, our results showed HR of N1a was similar to that of N1b. Additionally, changes in the HR for T2–3 and T4a-T4b were less significant than the HR for any other stage ordinal factor. PDTC presented more frequently with locally invasive extrathyroidal disease compared with DTC (25), and the Memorial Sloan Kettering Cancer Center (MSKCC) cohort study (11) showed that 59% of patients with gross T4a disease developed distant metastasis, indicating a strong correlation between the extent of ETE and the rate of distant metastases. Our study showed 23.6% of patients with T4 disease (48/203) in SEER cohort and 42% of patients with T4 disease (11/26) in China cohort had distant metastases. The patients with T4a-4b were 8 times more likely to death of disease compared to those with T1 disease due to the high rate of distant metastases of T4 disease.

In the 8th edition, the stage IVB patients with distant metastases include old patients with M1 disease irrespective of the T or N stages. By contrast, stage IVB in the new staging groupings includes old patients with T4 and M1 disease irrespective of N stages. In addition, the new staging groupings recommended that old patients with M1 disease and without T4 disease (44/876), old patients with T4 and N1 disease and without M1 disease (64/876) as well as young patients with M1 disease (15/876) be classified as having stage IVA disease. It is worth noting that only a small number of patients under the age of 55 had distant metastases (15 out of 82). Furthermore, older patients with T4 disease and without M1 disease had a high rate of lymph node metastasis (83.1% or 64 out of 77 patients). The new staging system’s introduction of stage IVA highlights the significant impact of T4 disease and age on the prognosis of PDTC.

The proposed staging system changes represented a significant departure from AJCC 8th staging system. Compared to the 8th edition, the new staging system appears to have a stronger correlation with CSS and demonstrates better monotonicity, discriminatory ability, balance of patient numbers, and predictive ability.

However, this study has several limitations. Firstly, the data from the SEER database were collected before the widespread implementation of the Turin criteria in 2007, potentially affecting the consistency in the identification of pathological subtypes. Secondly, due to the rarity of PDTC and the limited availability of detailed data, subgroup analyses within the proposed staging system were not conducted. Thirdly, the internal validation cohort, comprising patients diagnosed between 2014 and 2016 using the AJCC 8th edition, had a relatively short median follow-up time (63 months) and a limited sample size, which may affect the staging system’s stratification capability. Additionally, although external validation was performed using data from multiple high-volume institutions in China, we explicitly acknowledge that this external cohort had a relatively small sample size (n=85) and an uneven distribution across different stages, potentially limiting the validity and generalizability of the external validation results. Further validation in larger, more diverse, and age-balanced international cohorts is essential to confirm the generalizability and robustness of our staging system. Finally, while our study employed clinically established predictors validated through rigorous internal and external procedures, we acknowledge that penalized regression techniques (e.g., LASSO or Elastic Net) could help mitigate potential overfitting, especially in models involving multiple covariates. Incorporating these techniques in future research could further enhance the methodological rigor and predictive reliability of the staging system.

## Conclusion

Utilizing the SEER cohort, our study identified that the AJCC 8th staging system possess homogeneity in prognosis prediction. The developed TNM staging system for PDTC with a multicenter cohort for external validation demonstrated superior stratification and prognosis prediction.

## Data Availability

The raw data supporting the conclusions of this article will be made available by the authors, without undue reservation.
