# Supplementary material for: Development of a new TNM staging system for poorly differentiated thyroid carcinoma: a multicenter cohort study
Source: Front Endocrinol (Lausanne). 2025 Aug 19;16:1586542. doi: 10.3389/fendo.2025.1586542 (PMC12401677; doi:10.3389/fendo.2025.1586542)
Supplement: Supplementary file 1 [file DataSheet1.docx]

**Appendix Figure legends**

**Appendix Fig 1.** **Flow Chart Detailing the Selection of the Patients in This Study.**

**Appendix Fig 2.** **Decision‑Tree Flowchart for Assigning PDTC Subgroups to Stages I–IVB**

**Appendix Fig 3. Distribution of Hazard Ratios for 5-year Cancer-Specific Survival.** Hazard ratios were determined in the training cohort for both the (A) AJCC 8th staging, and (B) new staging. Stage I disease was used as the referent category.

**Appendix Fig 4.** **Staging Percentage Distribution for the AJCC 8th and New Staging System in Training Cohort**

**Appendix Fig 5**. **Consistency of Hazard Ratios for 5-year Cancer-Specific Survival for the Staging Subgroups of AJCC 8th and New Staging.** The subgroup of stage I; age ≥ 55 T1N0M0, was used as the referent category

**Appendix Fig 6. Schoenfeld Residual Tests for Proportional Hazards Assumption of Key Covariates (Age, T stage, N stage, and M stage).**

**Appendix Tables**

**Appendix Table 1. Patient Characteristics in the Training and Internal validation Cohorts from SEER Database**

**Appendix Table 2. Univariate and Multivariate Cox Regression Analyses of PDTC CSS**

**Appendix Table 3.** **The Probability of 5-year CSS of 24 Subgroups were Rearranged from High to Low**

**Appendix Table 4.** **The χ2 statistic on Cancer-Specific Survival for** **the AJCC 8th and New Staging System in Training Cohort**

**Appendix Table 5.** **Performance Evaluation of AJCC 8th and New Staging System using the Methodology of Bootstrap Algorithm by Xie et al in the Training** **Cohort**

**Appendix Table 6. Hazard Ratios of 5-year Cancer-Specific were Determined for the Staging Subgroups of AJCC 8th and New Staging in the Training Cohort.** The subgroup of stage I; age ≥ 55 T1N0M0, was used as the referent category

**Appendix Fig 1.**


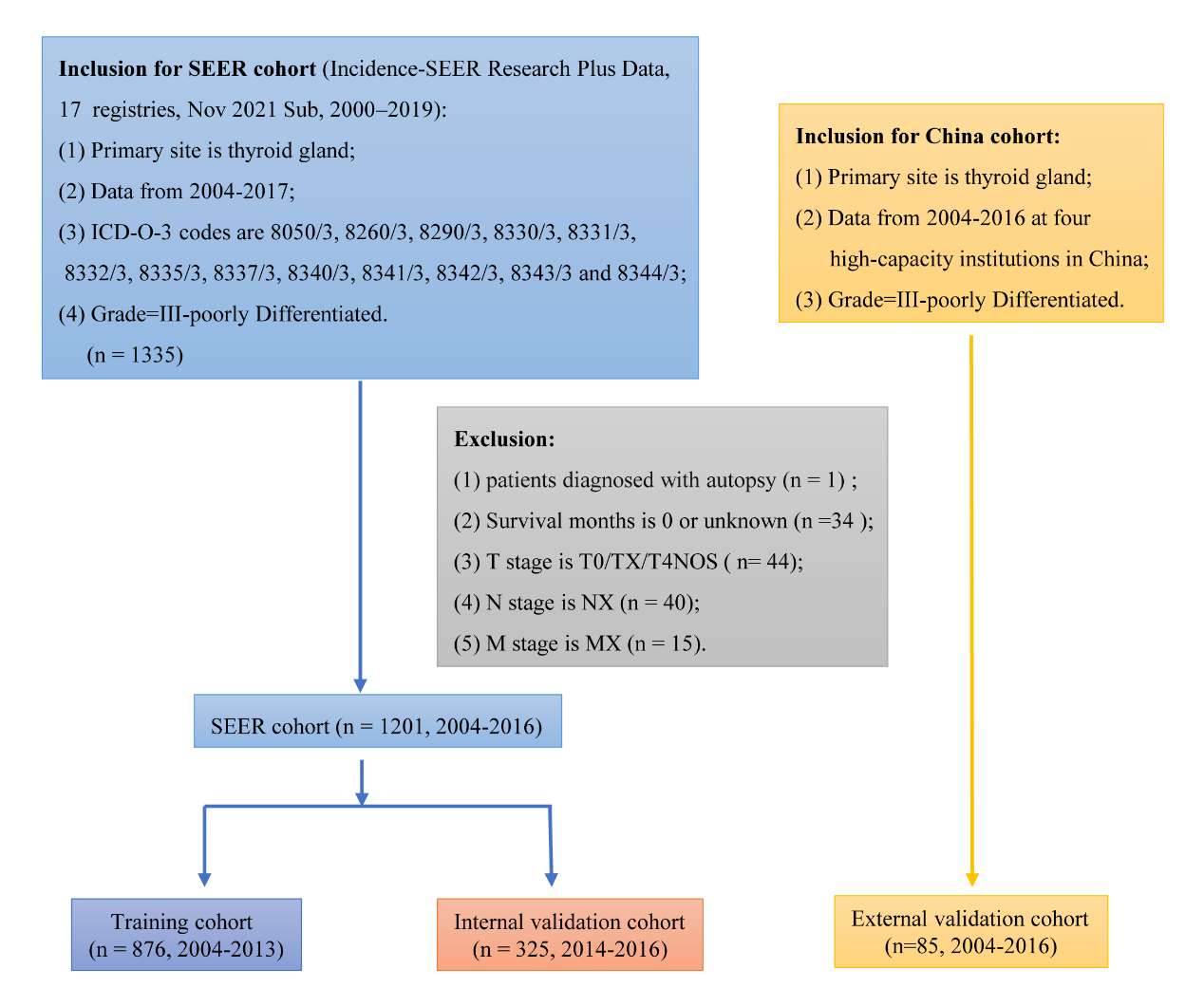


**Appendix Fig 2.**


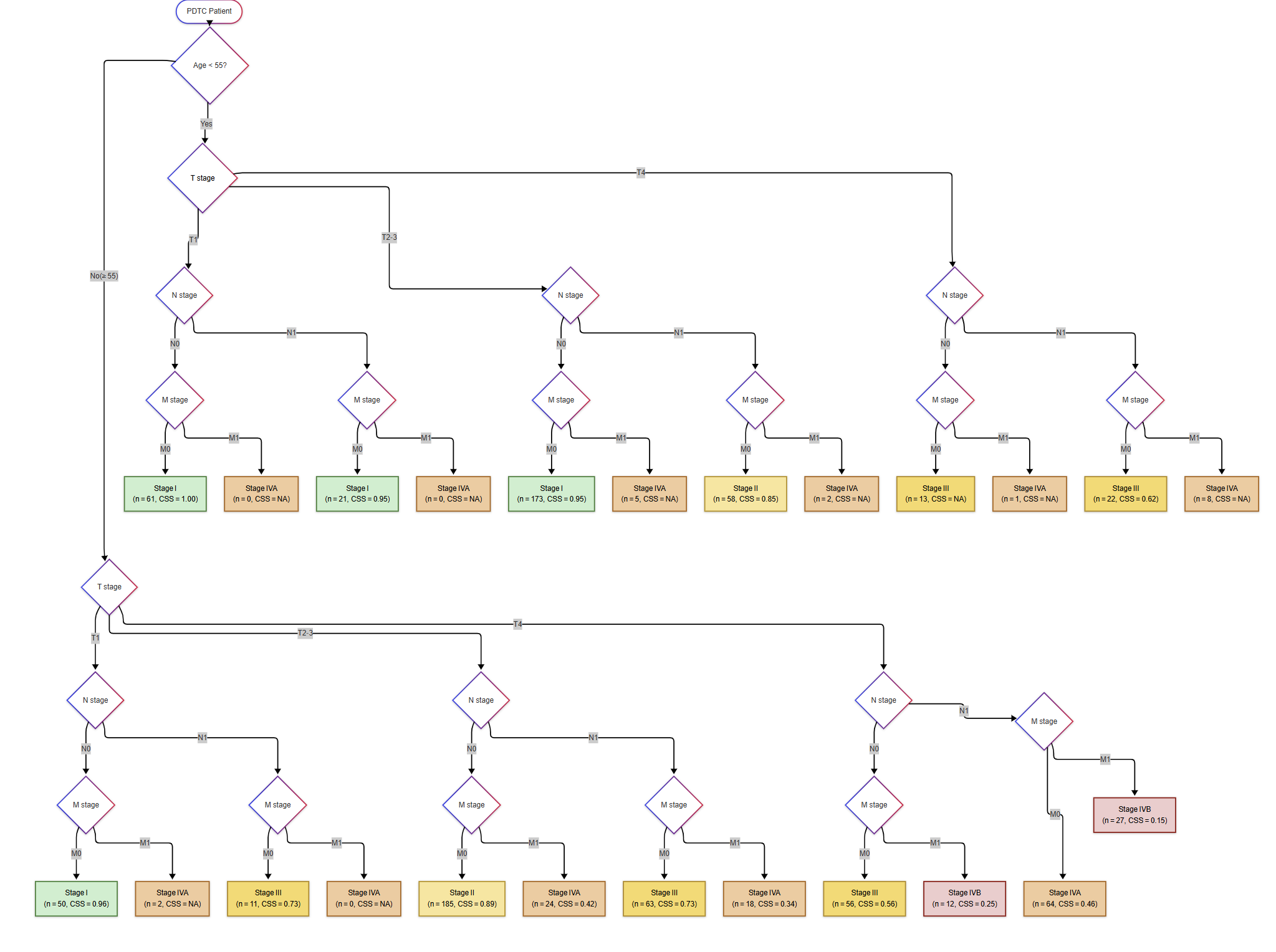


**Appendix Fig 3.**


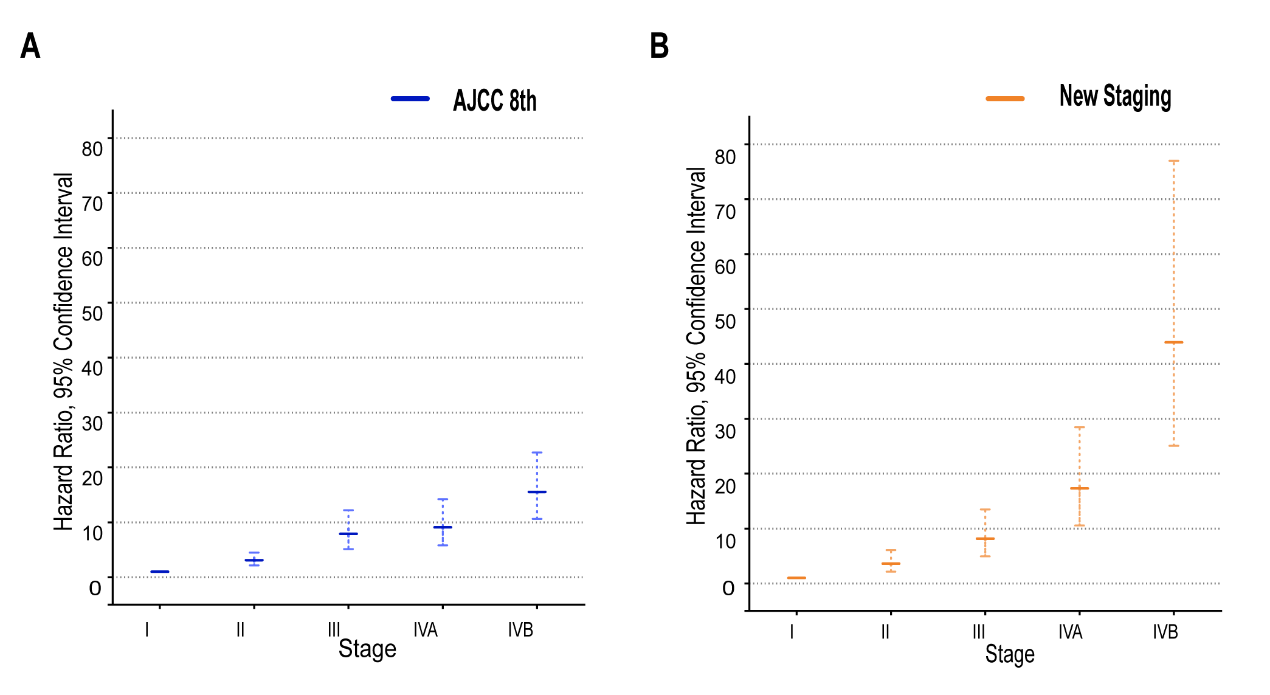


**Appendix Fig 4.**


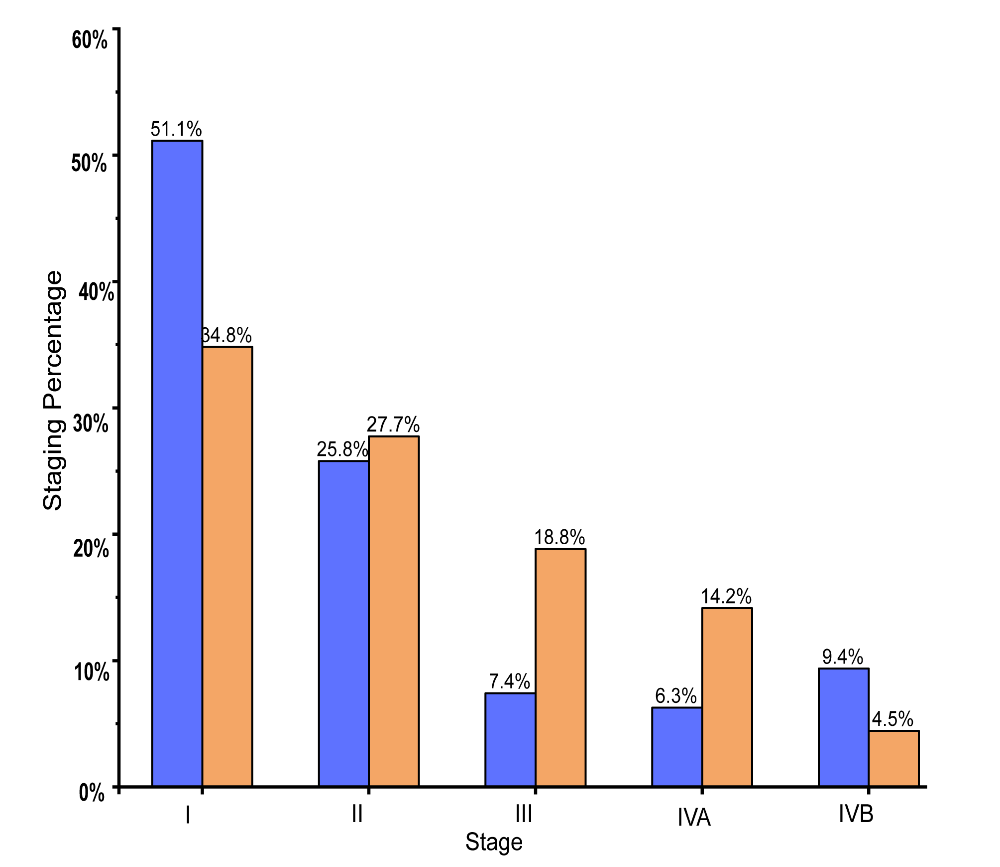


**Appendix Fig 5**.


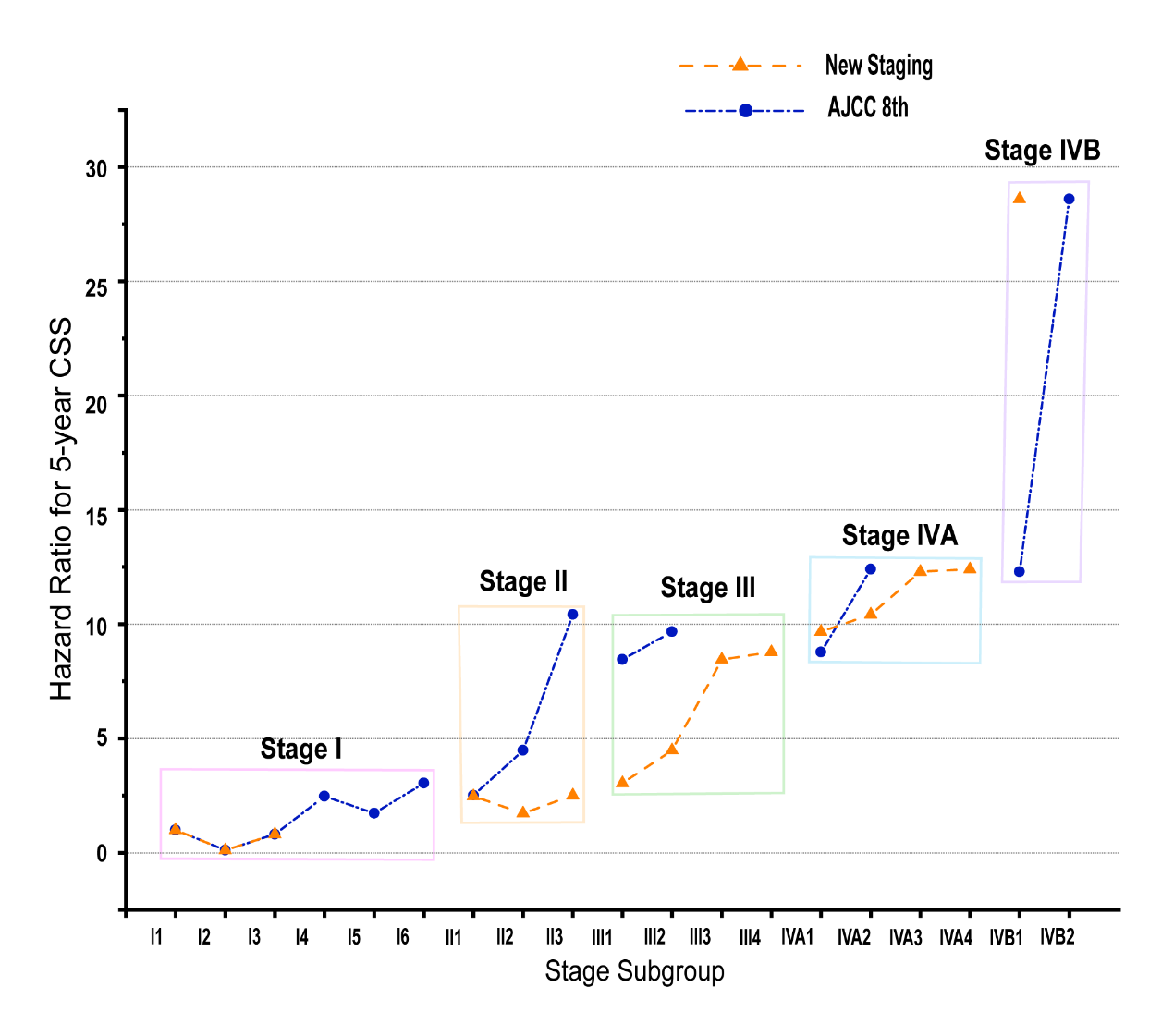


**Appendix Fig 6.**


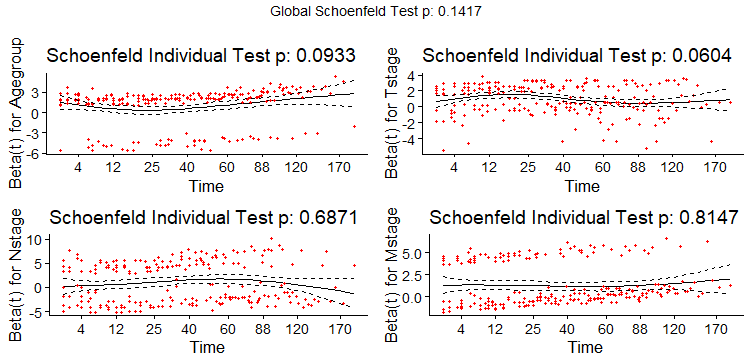


**Appendix Table 1.** Patient Characteristics in the Training and Internal Validation Cohorts from SEER Database

|  | **SEER overall**  **(n = 1201)** | **SEER 2004-2013**  **Training (n = 876)** | **SEER 2014-2016**  **Internal validation**  **(n = 325)** | ***P*** |
| --- | --- | --- | --- | --- |
| **Age, years** |  |  |  |  |
| < 55 | 512 (42.6%) | 365 (41.7%) | 147 (45.2%) | 0.297 |
| ≥ 55 | 689 (57.4%) | 511 (58.3%) | 178 (54.8%) |  |
| **Sex** |  |  |  |  |
| Female | 718 (59.8%) | 526 (60.0%) | 192 (59.1%) | 0.812 |
| Male | 483 (40.2%) | 350 (40.0%) | 133 (40.9%) |  |
| **Race** |  |  |  |  |
| Asian or Pacific Islander | 136 (11.3%) | 102 (11.6%) | 34 (10.5%) | 0.855 |
| Black | 99 (8.2%) | 70 (8.0%) | 29 (8.9%) |  |
| White | 952 (79.3%) | 693 (79.1%) | 259 (79.7%) |  |
| Other | 14 (1.2%) | 11 (1.3%) | 3 (0.9%) |  |
| **T stage** |  |  |  |  |
| T1 | 192 (16.0%) | 145 (16.6%) | 47 (14.5%) | 0.154 |
| T2 | 219 (18.2%) | 151 (17.2%) | 68 (20.9%) |  |
| T3 | 525 (43.7%) | 377 (43.0%) | 148 (45.5%) |  |
| T4a | 153 (12.7%) | 112 (12.8%) | 41 (12.6%) |  |
| T4b | 112 (9.3%) | 91 (10.4%) | 21 (6.5%) |  |
| **N stage** |  |  |  |  |
| N0 | 807 (67.2%) | 582 (66.4%) | 225 (69.2%) | 0.186 |
| N1a | 134 (11.2%) | 100 (11.4%) | 34 (10.5%) |  |
| N1b | 220 (18.3%) | 159 (18.2%) | 61 (18.8%) |  |
| N1NOS | 40 (3.3%) | 35 (4.0%) | 5 (1.5%) |  |
| **M stage** |  |  |  |  |
| M0 | 1054 (87.8%) | 777 (88.7%) | 277 (85.2%) | 0.126 |
| M1 | 147 (12.2%) | 99 (11.3%) | 48 (14.8%) |  |
| **Surgery** |  |  |  |  |
| Yes | 1171 (97.5%) | 853 (97.4%) | 318 (97.8%) | 0.797 |
| No | 30 (2.5%) | 23 (2.6%) | 7 (2.2%) |  |
| **Radiation** |  |  |  |  |
| Yes | 822 (68.4%) | 603 (68.8%) | 219 (67.4%) | 0.681 |
| No | 379 (31.6%) | 273 (31.2%) | 106 (32.6%) |  |
| **CSS** |  |  |  |  |
| Alive | 912 (75.9%) | 633 (72.3%) | 279 (85.8%) | < 0.001 |
| Dead | 289 (24.1%) | 243 (27.7%) | 46 (14.2%) |  |

Abbreviations: CSS, cancer-specific survival.

**Appendix Table 2.** Univariate and Multivariate Cox Regression Analyses of PDTC CSS.

| **Factor** | **Univariate analysis** | |  | **Multivariate analysis** | |
| --- | --- | --- | --- | --- | --- |
|  | **HR (95% CI)** | ***P*** |  | **HR (95% CI)** | ***P*** |
| **Age** |  |  |  |  |  |
| < 55 | 1 [Reference] |  |  | 1 [Reference] |  |
| ≥ 55 | 5.54 (2.75-11.16) | < 0.001 |  | 2.81 (2.01-3.93) | < 0.001 |
| **Sex** |  |  |  |  |  |
| Female | 1 [Reference] |  |  | 1 [Reference] |  |
| Male | 1.28 (1.00-1.65) | 0.055 |  | 0.91 (0.70-1.19) | 0.493 |
| **Race** |  |  |  |  |  |
| Asian or Pacific Islander | 1 [Reference] |  |  | 1 [Reference] |  |
| Black | 4.30 (0.22-0.85) | 0.015 |  | 0.92 (0.45-1.85) | 0.807 |
| White | 8.35 (0.58 -1.20) | 0.330 |  |  |  |
| **T stage** |  |  |  |  |  |
| T1 | 1 [Reference] |  |  | 1 [Reference] |  |
| T2 | 2.61 (1.16-5.90) | 0.021 |  | 2.25 (1.00-5.10) | 0.051 |
| T3 | 5.53 (2.69-11.37) | < 0.001 |  | 4.07 (1.97 -8.41) | < 0.001 |
| T4a | 15.94 (7.61-33.38) | < 0.001 |  | 8.81 (4.14 -18.79) | < 0.001 |
| T4b | 21.45 (10.22-45.01) | < 0.001 |  | 8.64 (4.02 -18.61) | < 0.001 |
| **N stage** |  |  |  |  |  |
| N0 | 1 [Reference] |  |  | 1 [Reference] |  |
| N1a | 2.05 (1.42-2.97) | < 0.001 |  | 1.84 (1.26-2.68) | 0.001 |
| N1b | 3.16 (2.37-4.22) | < 0.001 |  | 1.60 (1.17-2.20) | 0.003 |
| N1NOS | 2.05 (1.13-3.72) | 0.018 |  | 1.23 (0.66-2.28) | 0.513 |
| **M stage** |  |  |  |  |  |
| M0 | 1 [Reference] |  |  | 1 [Reference] |  |
| M1 | 6.49 (4.83-4.89) | < 0.001 |  | 3.61 (2.67-4.87) | < 0.001 |
| **Surgery** |  |  |  |  |  |
| Yes | 1 [Reference] |  |  | 1 [Reference] |  |
| No | 3.95 (2.30-6.79) | < 0.001 |  | 2.62 (1.47-4.64) | 0.001 |
| **Radiotherapy** |  |  |  |  |  |
| Yes | 1 [Reference] |  |  |  |  |
| No | 0.84 (0.63-1.12) | 0.235 |  |  |  |

Abbreviations: HR, hazard ratio; CI, confidence interval.

**Appendix Table 3.** The Probabilities of 5-year CSS of 24 Subgroups were Rearranged from High to Low.

| **Stage** | **Subgroups**  **(n = 24)** | **No.** | **5-year CSS** | **Sections**  **(n = 5)** |
| --- | --- | --- | --- | --- |
| **I** | Age < 55 T1 N0 M0 | 61 | 1.00 | 0.9-1 |
|  | Age ≥ 55 T1 N0 M0 | 50 | 0.96 |  |
|  | Age < 55 T2-3 N0 M0 | 173 | 0.95 |  |
|  | Age < 55 T1 N1 M0 | 21 | 0.95 |  |
| **II** | Age ≥ 55 T2-3 N0 M0 | 185 | 0.89 | 0.75-0.9 |
|  | Age < 55 T2-3 N1 M0 | 58 | 0.85 |  |
| **III** | Age ≥ 55 T1 N1 M0 | 11 | 0.73 | 0.55-0.75 |
|  | Age ≥ 55 T2-3 N1 M0 | 63 | 0.73 |  |
|  | Age < 55 T4 N1 M0 | 22 | 0.62 |  |
|  | Age ≥ 55 T4 N0 M0 | 56 | 0.56 |  |
|  | Age < 55 T4 N0 M0 | 13 | NA |  |
| **IVA** | Age ≥ 55 T4 N1 M0 | 64 | 0.46 | 0.30-0.55 |
|  | Age ≥ 55 T2-3 N0 M1 | 24 | 0.42 |  |
|  | Age ≥ 55 T2-3 N1 M1 | 18 | 0.34 |  |
|  | Age < 55 T4 N1 M1 | 8 | NA |  |
|  | Age < 55 T2-3 N0 M1 | 5 | NA |  |
|  | Age ≥ 55 T1 N0 M1 | 2 | NA |  |
|  | Age < 55 T2-3 N1 M1 | 2 | NA |  |
|  | Age < 55 T4 N0 M1 | 1 | NA |  |
|  | Age < 55 T1 N0 M1 | 0 | NA |  |
|  | Age < 55 T1 N1 M1 | 0 | NA |  |
|  | Age ≥ 55 T1 N1 M1 | 0 | NA |  |
| **IVB** | Age ≥ 55 T4 N0 M1 | 12 | 0.25 | 0-0.30 |
|  | Age ≥ 55 T4 N1 M1 | 27 | 0.15 |  |

Abbreviations: CSS, cancer-specific survival; NA, not applicable.

The 5-year CSS rate could not be calculated for the subgroups with a small number of patients, which were divided according to the principle of similar hazard ratio.

**Appendix Table 4.** The Difference in Cancer-specific Survival for the AJCC 8th and New Staging System in Training Cohort

|  | **AJCC 8th staging system** | |  | **The new staging system** | |
| --- | --- | --- | --- | --- | --- |
|  | **χ2 statistic** | ***P*** |  | ***P* for 5-year CSS** | ***P* for HR** |
| **I vs II** | 52.3 | 4.84E-13 |  | 28.7 | 1.17E-11 |
| **II vs III** | 19.2 | 1.19E-05 |  | 26.8 | 1.17E-05 |
| **III vs IVA** | 1.6 | 0.210 |  | 15.7 | 0.224 |
| **IVA vs IVB** | 5.3 | 0.022 |  | 28.2 | 0.054 |

Abbreviations: CSS, cancer-specific survival; HR, hazard ratio.

**Appendix Table 5.** Performance Evaluation of AJCC 8th and New Staging System using the Methodology of Bootstrap Algorithm by Xie et al in the Training Cohort.

| **Evaluation Criteria** | **AJCC 8th staging system** | **The new staging system** |
| --- | --- | --- |
| Hazard Consistency | 10.46 ± 14.56 | 13.00 ± 25.70 |
| Score | 0.42 ± 0.50 | 0.58 ± 0.49 |
| Rank | 1.42 | 1.576 |
| Hazard Discrimination | 1.20 ± 0.56 | 1.36 ± 0.61 |
| Score | 0.62 ± 0.48 | 0.38 ± 0.48 |
| Rank | 1.62 | 1.38 |
| Balance | 0.74 ± 0.03 | 0.45 ± 0.03 |
| Score | 1.00 ± 0.00 | 0.00 ± 0.00 |
| Rank | 2.00 | 1 |
| Outcome prediction (PVE) | 0.38 ± 0.02 | 0.40 ± 0.02 |
| Score | 0.77 ± 0.42 | 0.23 ± 0.42 |
| Rank | 1.77 | 1.23 |
| Overall Score | 2.82 ± 0.82 | 1.18 ± 0.82 |
| Overall Rank | 1.68 | 1.06 |
| Times in Rank 1 | 325 | 940 |
| % Rank = 1 | 0.26 | 0.74 |

Abbreviations: AJCC, American Joint Committee on Cancer; PVE: percent variance explained; %: percent

**Appendix Table 6.** Hazard Ratios of 5-year Cancer-Specific were Determined for the Staging Subgroups of AJCC 8th and New Staging in the Training Cohort.

| **Stage** | **AJCC 8th** | |  | **New staging** | |
| --- | --- | --- | --- | --- | --- |
|  | **Stage Subgroup** | **HR** |  | **Stage Subgroup** | **HR** |
| Stage I | Age ≥ 55 T1N0M0 | 1.00 |  | Age ≥ 55 T1N0M0 | 1.00 |
|  | Age < 55 T1N0-1M0 | 0.11 |  | Age < 55 T1N0-1M0 | 0.11 |
|  | Age < 55 T2-3N0M0 | 0.81 |  | Age < 55 T2-3N0M0 | 0.81 |
|  | Age < 55 T2-3N1M0 | 2.47 |  |  |  |
|  | Age ≥ 55 T2N0M0 | 1.73 |  |  |  |
|  | Age < 55 T4N0M0 | 3.05 |  |  |  |
| Stage II | Age ≥ 55 T3N0M0 | 2.52 |  | Age < 55 T2-3N1M0 | 2.47 |
|  | Age ≥ 55 T1-2N1M0 | 4.48 |  | Age ≥ 55 T2N0M0 | 1.73 |
|  | Age < 55 M1 | 10.42 |  | Age ≥ 55 T3N0M0 | 2.52 |
| Stage III | Age ≥ 55 T4aN0M0 | 8.45 |  | Age < 55 T4N0M0 | 3.05 |
|  | Age ≥ 55 T4aN1M0 | 9.67 |  | Age ≥ 55 T1-2N1M0 | 4.48 |
|  |  |  |  | Age ≥ 55 T4aN0M0 | 8.45 |
|  |  |  |  | Age ≥ 55 T4bN0M0 | 8.78 |
| Stage IVA | Age ≥ 55 T4bN0M0 | 8.78 |  | Age ≥ 55 T4aN1M0 | 9.67 |
|  | Age ≥ 55 T4bN1M0 | 12.40 |  | Age < 55 M1 | 10.42 |
|  |  |  |  | Age ≥ 55 T4bN1M0 | 12.40 |
|  |  |  |  | Age ≥ 55 T1-3N0-1M1 | 12.30 |
| Stage IVB | Age ≥ 55 T1-3N0-1M1 | 12.30 |  | Age ≥ 55 T4N0-1M1 | 28.60 |
|  | Age ≥ 55 T4N0-1M1 | 28.60 |  |  |  |

Abbreviations: HR, hazard ratio.

The subgroup of stage I; age ≥ 55 T1N0M0, was used as the referent category
